# Novel microbiome dominated by *Arcobacter* during anoxic excurrent flow from an ocean blue hole in Andros Island, The Bahamas

**DOI:** 10.1371/journal.pone.0256305

**Published:** 2021-08-19

**Authors:** Deborah D. Iwanowicz, Robert B. Jonas, William B. Schill, Kay Marano-Briggs

**Affiliations:** 1 Eastern Ecological Science Center, United States Geological Survey, Kearneysville, West Virginia, United States of America; 2 Department of Environmental Science and Policy, George Mason University, Fairfax, Virginia, United States of America; Harvard University, UNITED STATES

## Abstract

Andros Island, The Bahamas, composed of porous carbonate rock, has about 175 inland blue holes and over 50 known submerged ocean caves along its eastern barrier reef. These ocean blue holes can have both vertical and horizontal zones that penetrate under the island. Tidal forces drive water flow in and out of these caves. King Kong Cavern has a vertical collapse zone and a deep penetration under Andros Island that emits sulfidic, anoxic water and masses of thin, mucoid filaments ranging to meters in length and off-white turbid water during ebb flow. Our objective was to determine the microbial composition of this mucoid material and the unconsolidated water column turbidity based on the concept that they represent unique lithoautotrophic microbial material swept from the cave into the surrounding ocean. Bacterial DNA extracted from these filaments and surrounding turbid water was characterized using PCR that targeted a portion of the 16S rRNA gene. The genus Arcobacter dominated both the filaments and the water column above the cave entrance. *Arcobacter nitrofigilis* and *Arcobacter* sp. UDC415 in the mucoid filaments accounted for as much as 80% of mapped DNA reads. In the water column *Arcobacter* comprised from 65% to over 85% of the reads in the depth region from about 18 m to 34 m. Bacterial species diversity was much higher in surface water and in water deeper than 36 m than in the intermediate zone. Community composition indicates that ebb flow from the cavern influences the entire water column at least to within 6 m of the surface and perhaps the near surface as well.

## Introduction

Oxygen deficient water masses occur in oceans and estuaries globally. Some are caused by cultural eutrophication and climate change [1–4], while others are associated with water masses isolated from surface reaeration and/or oxygenic photosynthesis, often by salinity stratification, and these often have sulfidic deep water [5–8]. The latter situation is typical of karstic sink holes, often referred to as “blue holes” [6,8] which can have stable water quality characteristics that make them natural laboratories for the study of microbial diversity and biochemistry across oxic/anoxic interfaces. The terrestrial and ocean sink holes and caves on Andros Island, The Bahamas provide this kind of stability for investigating the microbiomes and biochemistry of oxygen depleted environments. By contrast estuarine and coastal ocean systems such as the Chesapeake Bay and Gulf of Mexico that develop anoxia seasonally now, but not in the past, are subject to weather and climate driven interannual variability [1–3,9]. Characterizing microbial communities as well as chemical, nutrient and biochemical dynamics across oxic/anoxic interfaces is enhanced by studying these natural laboratories.

**Fig 1:**
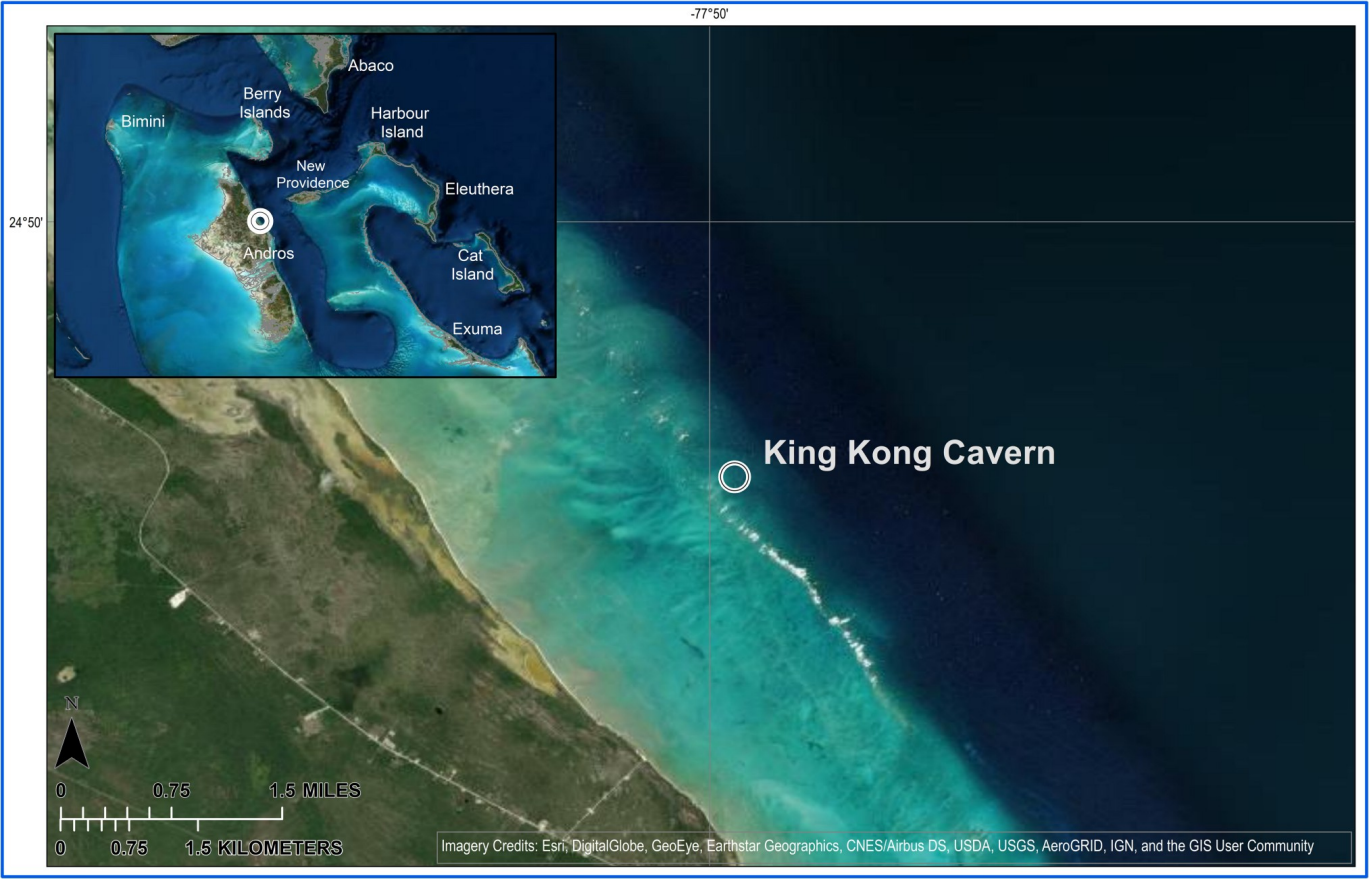
Map of Andros Island, The Bahamas showing the location of King Kong Cavern. Base map from World Imagery. Imagery Credits Esri, Digital Globe, GeoEye, Earthstar Geographics, CNES/Airbus DS, USDA, USGS and the GIS User Community.

These blue holes, often referred to as the “Eyes of Andros”, dot its surface, and unseen submarine caves lie along its east-facing barrier reef (Fig 1). In the ebb tide, outflow from one of these submerged caves, King Kong Cavern, there are masses of mucoid filaments in the water column and off-white turbid water below about 20 m. There is also a strong sulfide odor in the deep water but not at the surface. The presence of these filaments and the sulfide odor are predictably related to excurrent flow and disappear during flood tide. Because of very high sulfide concentrations, up to 18 mM, in the deep, anoxic water of a local terrestrial blue hole [10], and the karstic composition of Andros Island it was reasoned that sulfide oxidation and lithoautotrophy in King Kong Cavern could give rise to an especially abundant microbial community that might form these mucoid filaments and cause the visual turbidity in the water column. Since the microbial composition of the filaments and turbidity was unknown we collected environmental DNA to investigate the microbiome of these filaments and the planktonic community in the water column.

Andros, the largest island of the Commonwealth of The Bahamas at approximately 6,000-km^2^ land mass (160 km long, 64 km wide), is composed of karst carbonate rock derived from fossil coral and oolitic limestone [11]. There are approximately 175 inland blue holes, and about 50 more caves with entrances below sea level along the barrier reef of Andros. These submerged caves, referred to as ocean blue holes, often have both vertical and horizontal zones, the latter of which penetrate under the island itself. Both inland and ocean blue holes were exposed to air during past ice ages when sea level was at least 100 m below present. Ocean blue holes appear to be of two origins. Fracture zones along the eastern escarpment likely created some of these, while others, which are atop the reef structure, were likely formed by dissolution of the carbonate rock. An indication of the latter origin is a collapsed rubble pile at the entrance of our study site at King Kong Cavern.

The anchialine inland systems, such as Church’s Blue Hole (24° 44.529’ North x 77° 51.716’ West), are salinity stratified with oxic fresh or brackish water overlying an anoxic, sulfidic, high salinity hypolimnion [6,10,12]. The halocline of inland blue holes is maintained by lack of significant wind mixing, stable tropical temperatures and especially the seawater which flows slowly beneath and surrounds the island [7,13]. Hydrogen sulfide concentrations up to 18 mM [10] provide the electron donor supporting anoxygenic photosynthesis and chemoautotrophic primary production [6,14]. Investigations of other cave systems indicate that food webs can be supported by indigenous bacterial primary production [15,16].

Ocean blue holes are affected by lunar tides with significant inflow on rising tides and outflow on ebb tides. The tidal range along the Andros Barrier reef is modest at about 1 m. Nevertheless, the vigorous incurrent flow can result in vortices in the overlying water to over 3 m deep in some confined fracture zone submerged caves (personal observation), [17]. Even in collapsed cavern ocean blue holes water flows vigorously. Warner and Moore [18] described the ocean blue hole systems at Andros Island as having an incurrent “suck” current wherein the water was at ambient sea water temperature and contained plankton, detritus and sediment. They indicated that the “blow” current (excurrent) is cooler than ambient sea water and can contain hydrogen sulfide.

**Fig 2:**
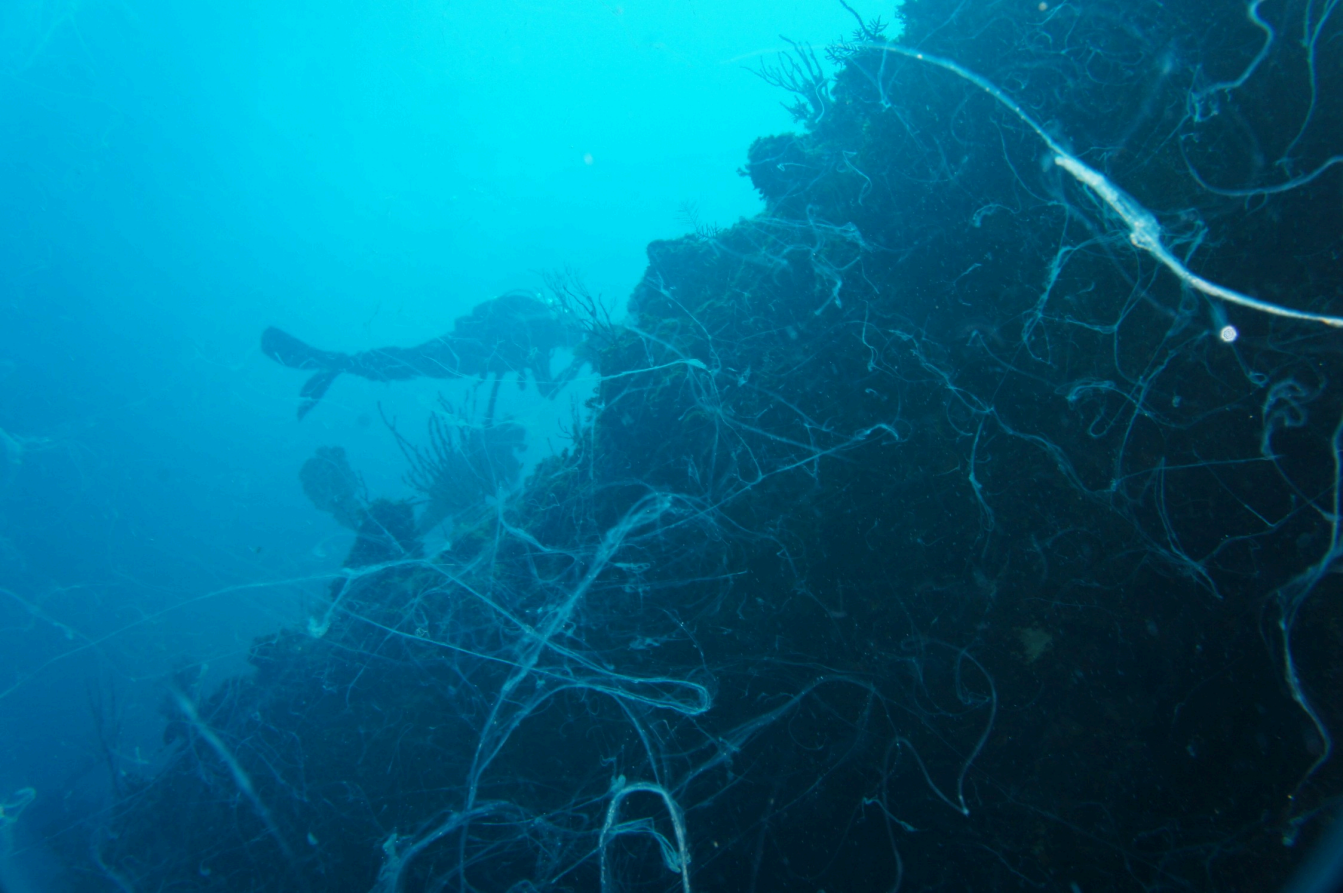
Mucoid filaments in the off-white turbidity zone of the water column. Image captured at approximately 25 m depth as viewed from below looking toward the surface (photo credit R.S. Jonas).

**Fig 3:**
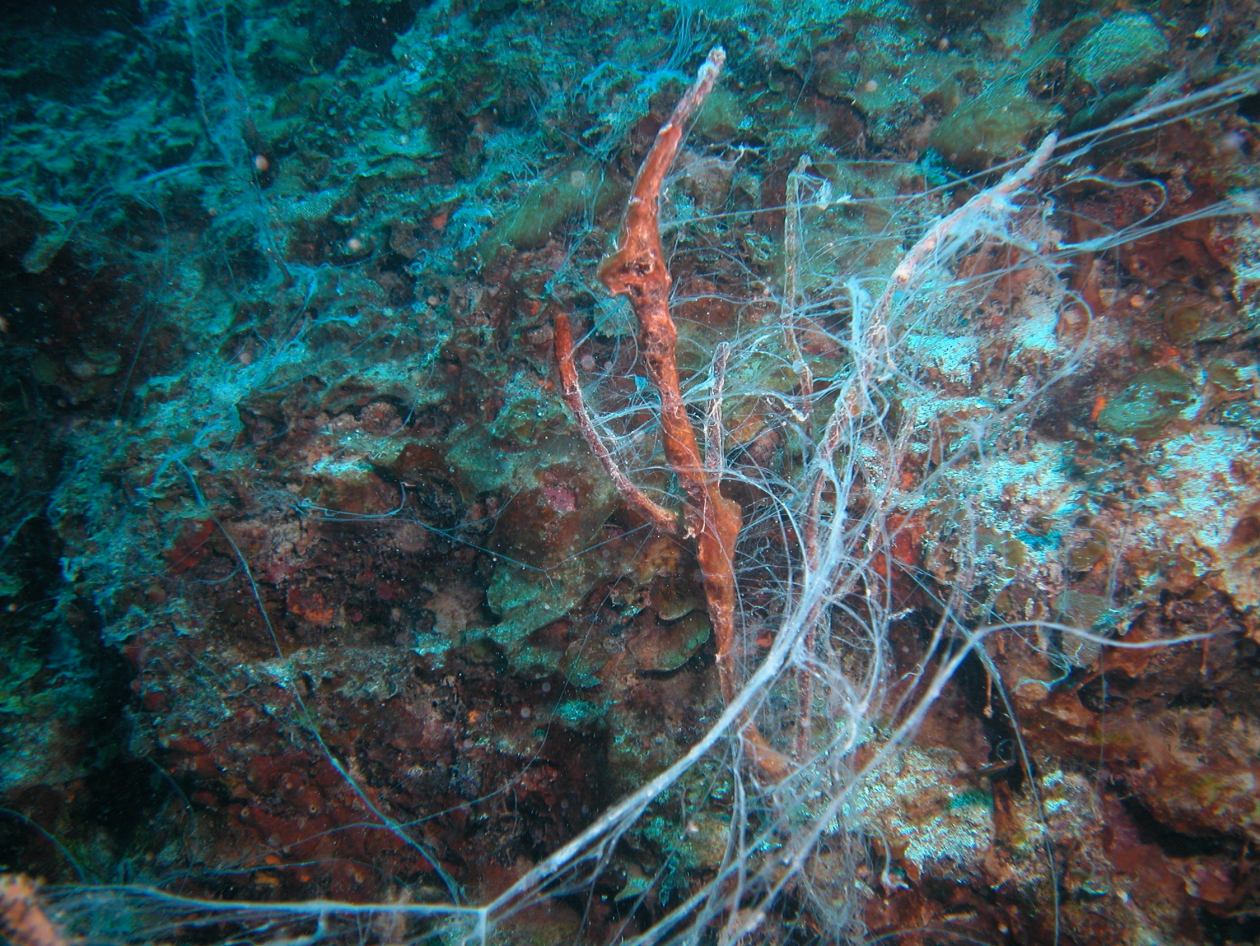
Mucoid filaments on reef structures surrounding King Kong Cavern. Image captured at approximately 15 m depth as viewed from above (photo credit R.B. Jonas).

During earlier visits to King Kong Cavern we observed this “suck and blow” cycle and the odor of hydrogen sulfide at depths below approximately 20 m. Most importantly we witnessed a layer of off-white, turbid water and large amounts of white mucoid filamentous material in the water column (Fig 2) at about 20 m and below and on reef structures (Fig 3) surrounding the cavern during the “blow” cycle. Based on our observation of high S^-2^ concentrations in inland blue holes and the porous nature of the carbonate rock we speculated that the hydrogen sulfide was derived from the inland blue holes and that sulfide fuels lithoautrophy within the ocean blue hole. Further, we reasoned that the off-white water column turbidity and the mucoid filamentous material were composed of bacteria and bacterial filaments swept off the cave walls and out of the cave during the high rates of excurrent flow.

Our principal goal in this research was to identify members of the microbiome of the excurrent (blow cycle) water column and the mucoid filaments found in King Kong Cavern using environmental DNA extraction and amplification. The current study focused on collecting samples at one stage of the tidal cycle. Future research should concentrate on the concept that chemoautotrophy within gives rise to high abundances of bacteria that are swept from the cave during excurrent flow. The work presented here is a first step in understanding the structure of this unique microbial ecosystem.

## Materials and methods

### Field collections

#### Sample site

Water and mucoid filament samples were collected at multiple depths from a karst sink hole (cavern) located along the Andros Barrier Reef. The sink hole, King Kong Cavern, is located at 24° 48.300’ North x 77° 49.512’ West approximately 3.6 km offshore (Fig 1). Water samples were collected under permit authorized by the Bahamas Environment, Science & Technology (BEST) Commission, Sydnei Cartwright, Environmental Officer. The westernmost edge of the cavern begins at approximately 18 m depth. Below that a vertical wall extends to about 23 m, at which point it angles downward toward the west to depths of 43 m. Beyond the cavern, the cave portion of this system extends to at least 143 m deep (Brian Kakuk, Bahamas Caves Research Foundation, personal communication).

#### Water column samples

On 28 June 2018 a reconnaissance dive by SCUBA confirmed the presence of white, mucoid filaments and an off-white turbid zone beginning at about 18 m depth and below (Fig 2) (S1 and S2 Videos). During previous observations at the site we found this turbidity and mucoid filaments during the excurrent water flow. On 01 July 2018 a water column profile of physicochemical parameters (YSI Pro Plus Multimeter) was measured at depths ranging from the surface to 30 m during the middle stage of the ebb tide. During the later stage of the ebb tide, bulk water and mucoid filament samples were collected by SCUBA diver using new, sterile, capped, 60 ml, all polyethylene/polypropylene syringes (S3 Video). All divers wore latex gloves during sample collection and handling. Syringes were affixed to a 1 m long PVC tube with a separate indwelling PVC tube which pulled the syringe plunger. The syringe caps were removed and the 1 m tube extended away from the diver, the samples were collected, and syringes immediately recapped. Eight general water samples, arrayed from 1.0 m to approximately 37 m, were collected along with three samples focused on the mucoid filaments floating in the water column, two at 18.3 m and one at 21.4 m. Samples were immediately returned to the research vessel and refrigerated until processing within approximately two hours.

#### Sample processing and storage

A filtration apparatus was thoroughly rinsed with 0.2 μm porosity filtered sea water prior to sample concentration. The 60 ml water samples and the mucoid samples were filtered through 0.2 μm porosity, polycarbonate filters under 7 in of mercury vacuum. The filters were then individually placed into 2.0 ml collection vials with microbe bashing beads (Zymo Research, Irvine, CA). The genomic DNA was preserved by using a pneumatic palm-nailer to homogenize filter-captured microbial cells in 800 μl of DNA/RNA Shield (Zymo Research, Irvine, CA). The preserved samples were then refrigerated until analyzed.

### DNA extraction and sequencing

#### DNA extraction from water samples

Genomic DNA, from the samples preserved in DNA/RNA shield, was extracted following the methods of the Zymo ZR Soil Microbe DNA Kit™, (Zymo Research, Irvine, CA). All extracted DNA was stored at -20°C until polymerase chain reaction (PCR) was performed.

#### Amplification of the 16S rRNA region

Bacteria were characterized using PCR that targeted a portion of the 16S rRNA gene. Amplicons were produced in two steps, first using standard primers to generate a high concentration of input template. This was followed by less efficient fusion primers that incorporate exogenous sequencing adapters that were used for the creation of full-length Illumina MiSeq sequencing libraries. Sequencing of 16S rRNA used primer pair sequences for the V3 and V4 region that create a single amplicon of ~460 base pairs (bp). The primers for the first amplification reaction were 16S For (5’–CCTACGGGNGGCWGCAG– 3’) and 16S Rev (5’–GACTACHVGGGTATCTAATCC– 3’) [19]. The thermocycler program consisted of an initial denaturation step of 95°C for 3 min, followed by 35 cycles of 30 s at 95°C, 30 s at 55°C, and 30 s at 72°C, for 7 minutes. An appropriately sized amplification product was confirmed for each reaction by electrophoresis of 5 μL of the reaction product through a 1.2% I.D.NA™ agarose gel (Cambrex Corporation, East Rutherford, NJ) at 100 V for 45 min. PCR products were cleaned with a QIAquick PCR Purification Kit (Qiagen CT # 28104, Valencia, CA) and quantified using the Qubit dsDNA HS Assay Kit (ThermoFisher Scientific, Grand Island, NY). Samples were diluted in 10 mM Tris buffer (pH 8.5) to a final concentration of 5 ng/μL.

#### Library preparation, quality assessment, and read filtering

Using the 16S rRNA primers described above modified with the sequencing overhangs specified in Illumina’s 16S Metagenomic Sequencing Library Preparation (CT #: 15044223 Rev. B), amplicon libraries were prepared following the manufacturer’s protocol. Each sample was indexed with Illumina’s Nextera XT multiplex library indices, which incorporates two distinct 8 bp sequences on each end of the fragment. Libraries were quantified with the Qubit dsDNA HS Assay Kit (ThermoFisher Scientific, Grand Island, NY). DNA size spectra were determined with the Agilent 2100 Bioanalyzer using the Agilent DNA 1000 Kit (Santa Clara, CA). Indexed libraries were diluted to 4 nM using 10 mM Tris pH 8.5 to normalize and pool by combining equal volumes. A final 10 pM preparation was created with a 15% PhiX control spike and run on a MiSeq 600 v3 cartridge. Simple on-instrument data analysis was performed with MiSeq Reporter software that is pre-installed on all MiSeq sequencers. This software processes base calls generated on-instrument during the sequencing run into fastq files. These machine-processed FASTQ files were imported into One Codex (San Francisco, CA) for alignments and classifications [20–22] and deposited into BioProject PRJNA703959, SRA accession SRP309919. Diversity indices, based on species composition, were calculated within the One Codex software.

## Results

### Physicochemical profile

**Fig 4:**
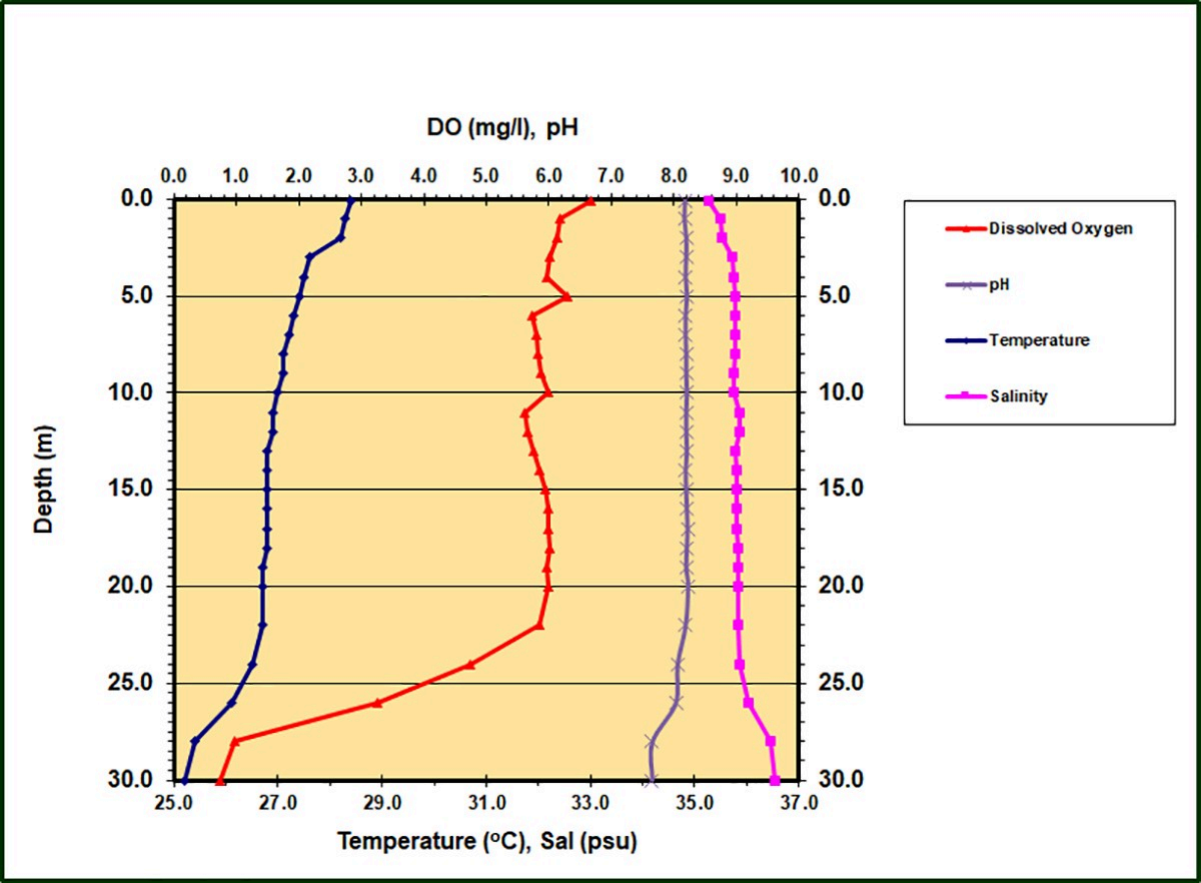
Depth profile of dissolved oxygen, pH, temperature and salinity in King Kong Cavern. Data collected during mid ebb flow from the cavern.

Water temperature was about 28°C at the surface and declined slowly with depth to about 25°C (Fig 4). There were two modest thermoclines in the water column, one at about 3 m and another, more intense one, at 27 m. These changes in temperature corresponded roughly to modest but detectable increases in salinity with depth in the upper water column and again in the depth range from 24 m to 28 m. Surface salinity was about 35 psu, while salinity at 30 m was about 36.5 psu. Although overall salinity changes were less than 1.5 psu, an especially notable halocline occurred below 24 m. Dissolved oxygen was saturated at the surface (6.7 mg l^-1^) and remained above 89% saturation to a depth of 22 m, with some small but noticeable variations within that depth range. From 22 m to 30 m there was a dramatic oxycline with values declining to 0.7 mg l^-1^ at 30 m. Water column pH remained stable at about 8.2 from the surface down to 22 m and then declined to 7.6 at 30 m. These data were gathered about 2 hours prior to collection of water samples. A slight but detectable characteristic odor of H_2_S was noted in samples from 24.4 m, and the intensity of that odor increased with increasing depth. No H_2_S odor was observed above 18 m.

### DNA sequencing

The total number of samples consisted of 8 water samples taken at varying depths within King Kong Cavern and 3 mucoid filament samples. The total read output for the 8 water samples was 11,169,956 after initial trimming based on quality and ambiguity adaptor trimming. The total read output for the 3 mucoid filament samples was 4,936,356 after initial trimming based on quality and ambiguity adaptor trimming. The genus *Arcobacter* dominated most all samples, comprising 59.6% of all reads for the water samples, and 55.4% of all reads for the mucoid filament samples. Most all *Arcobacter* reads were classified by One Codex as either *Arcobacter* sp. UDC415 or *Arcobacter nitrofigilis*. Machine processed sequencing output was deposited in NCBI’s Short Read Archive under BioProject PRJNA703959.

### Mucoid filament microbial composition

One of the most notable features of King Kong Cavern is the regular, predictable presence of large amounts of white, mucoid filaments in the water column during excurrent flow from the cave (Figs 2 and 3). Two replicate mucoid filament samples (KS1 and KS2) collected at 18.3 m and one (KS3) taken at 21.4 m all coalesced into compact balls in the collection syringes before filtration.

**Fig 5:**
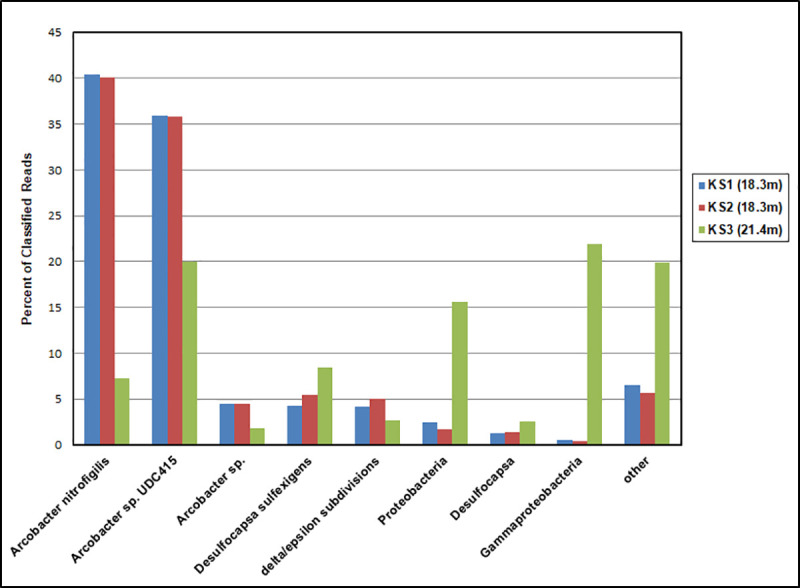
Major bacteria in mucoid filaments in the water column of King Kong Cavern. Species assignment from One Codex (San Francisco, Calif).

The seven most dominant genera/subgroupings in these samples as per One Codex are shown in Fig 5. Replicate samples KS1 and KS2 were dominated by the genus *Arcobacter* and the 16S sequences suggest two distinct types identified in One Codex as *A*. *nitrofigilis* and *Arcobacter* sp. UDC415. The genus *Arcobacter* represented 80.8% (KS1) and 80.3%% (KS2) of all reads in these mucoid filament communities. Within the One Codex results *A*. *nitrofigilis* represented 40% of all reads in KS1 and KS2 and *Arcobacter* sp. UDC415 represented 36% of all reads while unidentified members of the *Arcobacter* genus accounted for 4.4%.

*Arcobacter* were also abundant in the KS3 sample from 21.4 m with *Arcobacter* sp. UDC415 representing the single largest species component at 20% of all reads. Overall the genus *Arcobacter* represented about 29% of the total KS3 community. Unidentified proteobacteria and gammaproteobacteria together represented 37% of community composition in KS3 but only 2–3% in the KS1 and KS2 samples. *Desulfocapsa sulfexigens* along with other unidentified species of *Desulfocapsa* were found in all three samples and represented between 5% and 11% of all classified reads. The mucoid community sampled at 21.4 m was more diverse (Simpson D = 0.67, Shannon H = 2.8) than that collected at 18.3m (D = 0.19 and 0.20, H = 0.80 and 0.80) [23,24].

### Water column microbiome

**Fig 6:**
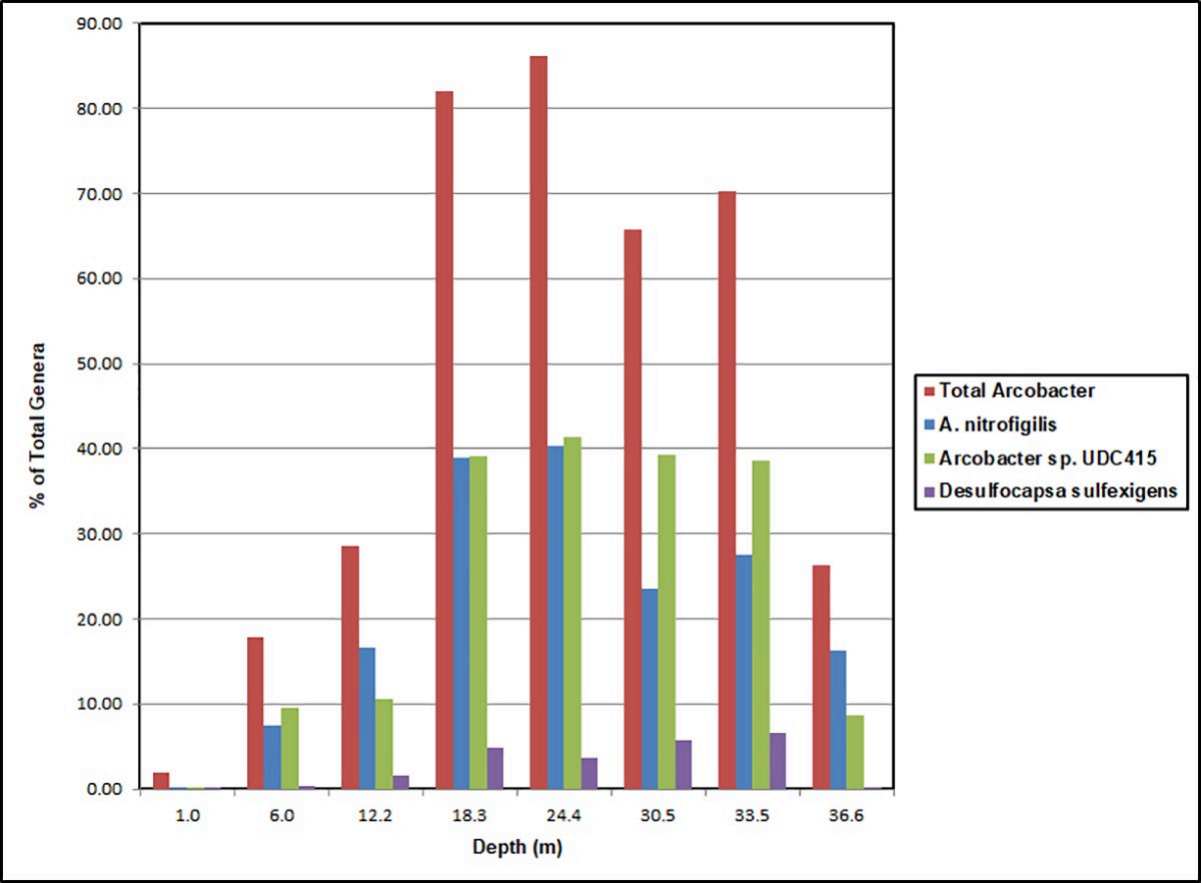
Percent of classified bacterial reads in the water column of King Kong Cavern. Data are shown for total *Arcobacter* at the genus level, and *A*. *nitrofigilis*, *Arcobacter* sp. UDC415 and *Desulfocapsa sulfexigens* at the species level as determined using One Codex (San Francisco, California).

*Arcobacter* were detected in all the water column samples (Fig 6). Both *A*. *nitrofigilis* and *Arcobacter* sp. UDC415 were present at all depths. However, at 1.0 m they were minor components, while *A*. *bivalviorum* was the most abundant *Arcobacter* species at 1.24% of classified reads. In all of the remaining samples from 6.0 m to 36.6 m, *A*. *nitrofigilis* and *Arcobacter* sp. UDC415 dominated the *Arcobacter* portion of the community and accounted for about 80% of all classified reads at depths from about 18 m to 25 m. At greater depths these *Arcobacter* species declined slightly in importance, but even at 36.6 m they still represented about 25% of all reads.

At depths from about 18 m to 34 m *Desulfocapsa sulfexigens* was the second most abundant species among classified reads representing 5% to 6.5% of the community. It was detected at very low abundance at 1.0 m (0.02%) and at 36.6 m (0.04%) and at about 1.5% at 12.2 m. *Desulfocapsa thiozym*ogenes and unidentified species of *Desulfocapsa* were also found. *D*. *thiozym*ogenes was less than 1% of classified reads while other *Desulfocapsa* were as high as 1.8%, and both were at their maxima between 18 m and 34m.

**Fig 7:**
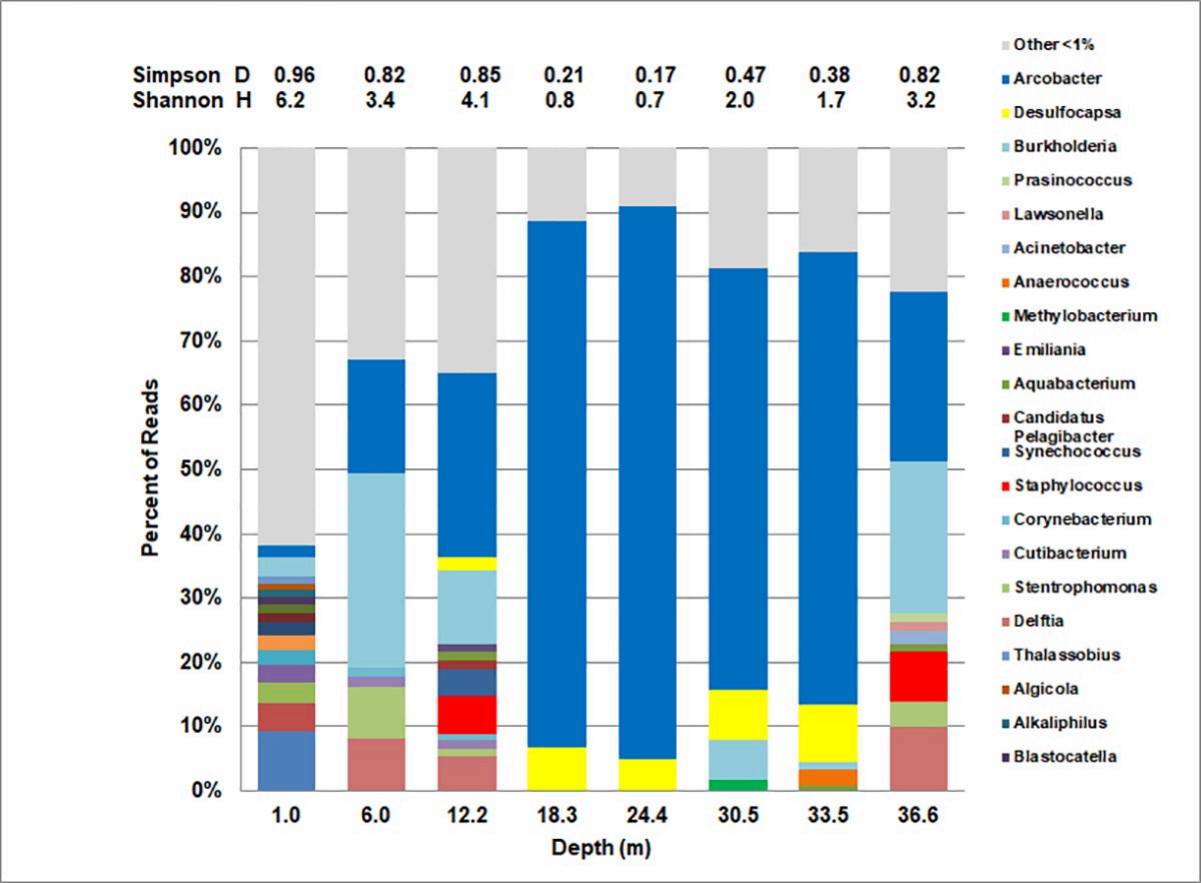
Distribution of major bacterial genera in the water column of King Kong Cavern. Community genus diversity indices are shown at the top of the figure (Simpson Diversity Index—D, Shannon Diversity Index–H).

Bacterial genera representing the highest percentages (more than 1.0%) of reads at multiple depths of King Kong Cavern are shown in Fig 7 along with Simpson (D) and Shannon (H) diversity indices. The microbiome at 1.0 m was the most diverse. Sixteen genera were present at greater than 1.0% of the reads, and 545 bacterial species and 201 genera with unidentified species were found in that sample. By comparison at 24 m there were only two genera, *Arcobacter* (86%) and *Desulfocapsa* (4%) with more than 1.0% of reads and only 127 species and 49 genera with unidentified species. Bacterial diversity was markedly lower in the mid-water between 18 m and 34 m than at shallower depths. However, diversity increased at 36.6 m and was comparable to that at 6 m. At both of those depths many of the same most abundant genera including *Burkholderia*, *Arcobacter*, *Delftia*, *Staphylococcus* and *Stenotrophomonas* were present. *Arcobacter* was by far the dominant genus between 18 m and 34 m, where *Desulfocapsa* had a much smaller presence but still represented from 4 to 6.5% of reads.

## Discussion

### Physicochemical parameters

The water column at King Kong Cavern above about 18 m was analytically and visually unremarkable. Below that zone, however, there were significant changes in water quality and visibility. During the ebb the fact that salinity increased slightly below 25 m indicates that the flow at that depth did not originate in the fresh to brackish upper portions of the inland anchialine blue holes or fresh groundwater, nor from the surrounding ocean water that flowed into the cavern on flood tide. Surface seawater salinity was about 35.5 psu, whereas salinity at 30 m was about 36.5 psu. Stringfield and LeGrand [25] in their assessment of karst features affecting water circulation in coastal carbonate rock formations suggested that on Andros Island the freshwater head is sufficient to create submarine springs along the shallow top of the escarpment bordering the east side of the island. Water quality characteristics from King Kong Cavern do not fit that scenario. Rather it seems likely that the relatively high salinity water at depth reflects the high salinity water found below about 25 m in some inland blue holes [6] or the slightly hypersaline water (ranging up to 42 psu) on the Great Bahama Banks [26].

Throughout the water column, dissolved oxygen remained above 90% saturation to a depth of 20 m and then declined rapidly along with a decline in pH of about 0.5 units. When water for genomic analyses was collected at these depths there was a slight hydrogen sulfide odor in the 24.4 m sample and strong hydrogen sulfide odor in all the samples below that. Hydrogen sulfide has been reported in ebb-flow water from other marine caves but not all [12]. Inland blue holes generally have a “sulfide” zone in the subpycnocline water [27]. The origin of the reduced sulfur is from bacterial sulfate reduction [28]. While we assume that the sulfide in the waters of King Kong Cavern is of bacterial origin, it remains to be determined if this is from sulfate reduction in the cavern itself, from subpycnocline waters of inland blue holes or of subterranean origin. Given the hypersaline water at depth in King Kong Cavern it seems doubtful that the sulfide derives directly from inland blue holes.

### Microbiome of mucoid filaments

The genus *Arcobacter* dominated the microbiome of the mucoid filaments floating in the water column. This genus represented over 80% of classified reads in KS1 and KS2 replicates and almost 30% in KS3. The filament samples evaluated for this work were collected at nearly the same time on a single ebb tide. The differences in microbial composition between the replicate KS1 and KS2 and the deeper KS3 sample may represent the dynamic sequence of bacteria swept from the cave on the ebb. Clearly a tidal cycle study is needed to explore the cause of differences in community composition. It remains to be determined if the mucoid filaments derive intact from the cave or are the result of coalescing of dispersed bacteria in the water column (e.g. the off-white turbidity).

*A*. *nitrofigilis* was the dominant species amplified from the mucoid filaments in samples KS1 and KS2. The observed water chemistry, specifically low oxygen concentration and the salt content, are consistent with its microaerophilic to anaerobic growth habit and salinity requirements [29,30]. In their assessment of the taxonomy of *Arcobacter* as currently recognized, Pérez-Cataluña et al. [30] indicate there are sufficient genetic differences within the genus to divide it into seven or more genera. Analysis of data submitted in BioProject PRJNA703959 indicated that 89.4% of reads were in the Arcobacter group and the Krona view [31] resulted in an average genus distribution of 23% *Arcobacter*, 58% *Malaciobacter* and 7.5% *Poseidonibacter*.

The second highest percentage of reads in KS1 and KS2 in the One Codex taxonomy were for an unnamed *Arcobacter* (*Arcobacter* sp. UDC415, NCBI:txid762480) which was isolated from the Dokdo Island, Korea in the Sea of Japan (East Sea) and accessioned in Genbank in 2010 (Ghim, S.Y. Cultivable bacterial diversity of Dokdo Island, Korea, unpublished) and again in 2012 (Sung, H.R. Bacterial diversity and distribution of cultivable bacteria isolated from Dokdo island, unpublished). In sample KS3, *Arcobacter* sp. UDC415 represented the largest percentage of *Arcobacter* reads.

*Arcobacter* sequences have been found from a variety of inland, freshwater, sulfidic caves specifically in various surface associated and filamentous biofilms [32,33], although *Arcobacter* was a relatively minor component in these caves. They have also been found to be a dominant member within sulfur mats at some hydrothermal vents [34]. He et al. [8] found high relative abundances (24%) of *Arcobacter* amplicons at about 100 m depth in the anoxic, sulfidic 300 m deep Sansha Yongle Blue Hole in the South China Sea.

We are unaware of any scientific reports documenting the presence and composition of mucoid filaments such as those in King Kong Cavern. D’Angeli et al. [35] describe floating and attached filaments in a partially sea water/ sulfuric acid cave along the Adriatic coast of Italy. Epsilonproteobacteria, including *Arcobacter*, were present in those filaments, but they were not the most dominant. Our dive team did not penetrate the actual cave portion of this system, reaching a maximum depth of only about 37 m. Although there appeared to be small (2 cm) patches of white filamentous material on the rock surfaces, we did not observe any large, whitish mats which might be the source of the mucoid filaments swept out of the cave on the ebb flow. It is possible such mats proliferate deeper in the cave. Another possibility is that the filaments are produced by coalescing of “planktonic” material within the cave during the excurrent flow. Although *A*. *nitrofigilis* has not been reported to produce sulfur filaments as does *A*. *sulfidicus* [36], investigation of its growth habits will be important. Similarly, *Arcobacter* sp. UDC415 should be investigated with regard to possible production of sulfur containing filaments. In any event it seems likely that bacterial primary production could be a major process within this marine blue hole with the excess production being carried out of the cave during high dynamic flow and potentially adding organic material to the surrounding reef system (Fig 3).

*Desulfocapsa sulfexigens*, present KS1/KS2 and in KS3, originally isolated from tidal flat [37], is an anaerobic chemolithotroph [38,39]. There is little information about its environmental importance, but data presented here suggest it may be ecologically important in King Kong Cavern.

In KS3, proteobacteria and especially gammaproteobacteria represented a substantial portion of the reads not classified at the genus level (Fig 5). Approximately 20% of the KS3 reads were unclassified. The microbiome of the KS3 sample was more diverse (D = 0.79, H = 3.5) than that in KS1/KS2 (D = 0.58, H = 1.6) at the species level. Since these mucus–like filaments were collected within minutes of one another and only about 3 m difference in depth, the difference in relative community composition is remarkable. Despite the single KS3 sample collected, the differences in microbiome composition may represent some tidally related dynamic physical process. A sampling regime over a tidal cycle that incorporates replicate sampling will be necessary to investigate these dynamics.

## Water column microbiome

At the time of sampling the top of a visibly turbid zone began at about 18 m. That zone contained both general off-white turbidity and the mucoid filaments. The percent of classified reads in the 18.3 m water column sample mirrored those in the focused filament samples KS1/KS2. *Arcobacter* dominated the classified reads with *A*. *nitrofigilis* and *Arcobacter* sp. UDC415 each accounting for about 40% of reads (Fig 6). In contrast, the bulk water microbiome from 24.4 m, with 80% *Arcobacter*, was significantly different from the mucoid filament sample KS3 from 21.4 m, with *Arcobacter* at only 29% of total reads. *Arcobacter* continued as the dominant genus of classified reads in the bulk water to the maximum depth sampled although it was just over 26% at 36.6 m (Fig 6). At that depth, *Burkholderia* was nearly as prominent at about 24% of reads. Above the turbid layer *Arcobacter* still represented a significant portion of the classified reads (28% at 12.2 m and about 18% at 6.0 m). This was considerably above the depth at which physicochemical data and visual observation would suggest that the surface zone was affected by ebb flow from the cavern itself.

**Fig 8:**
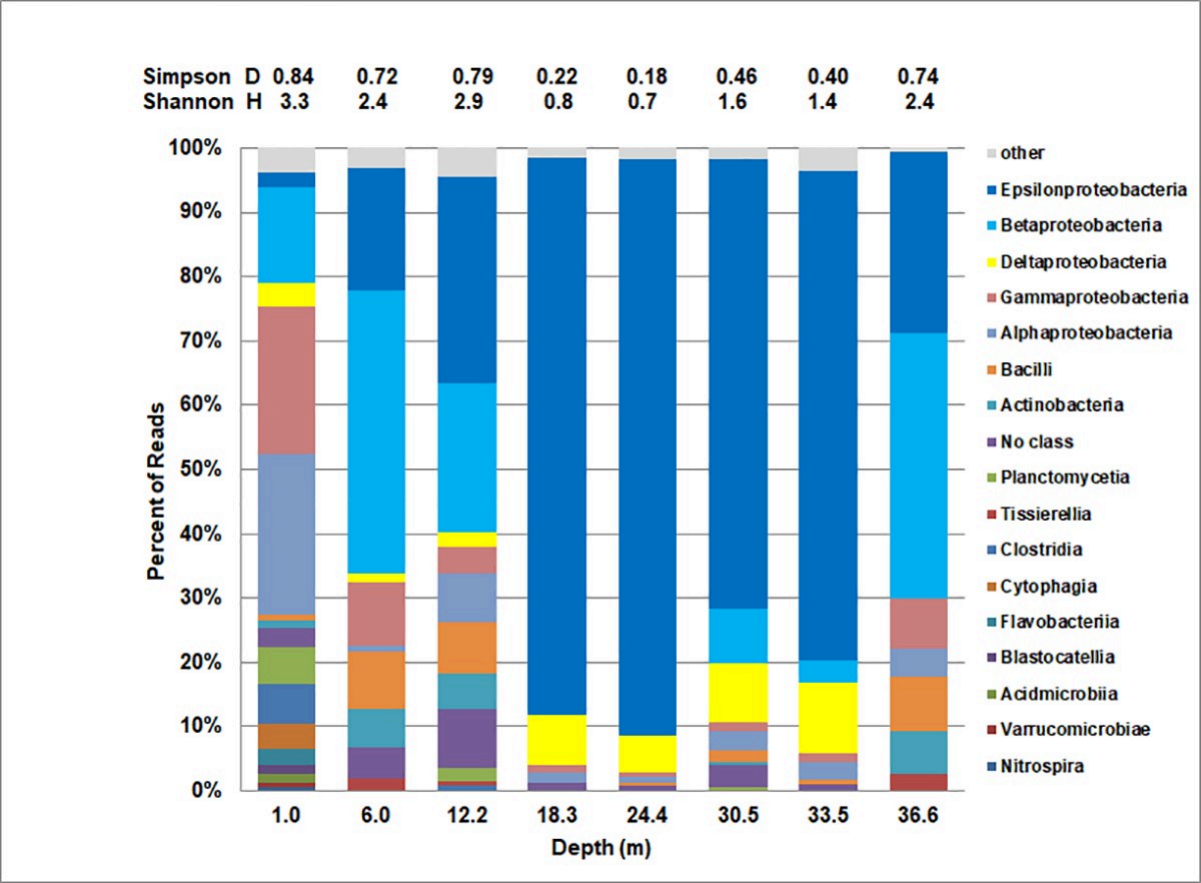
Distribution of major bacterial classes in the water column of King Kong Cavern. Bacterial class diversity indices are shown at the top of the figure (Simpson Diversity Index—D, Shannon Diversity Index–H).

At the Class level Epsilonproteobacteria (*Arcobacter* group) dominated the water column between approximately 18 m and 34 m (Fig 8). At approximately 36 m Betaproteobacteria were most prevalent at 41% (*Burkholderia*, *Delftia*) and Epsilonproteobacteria represented only 28%. Above the visibly turbid zone at 12 m the community was more diverse (D = 0.79, H = 2.9) than below, but Epsilonproteobacteria were still the single most abundant class (34%). The community at 6 m still had significant Epsilonproteobacteria present, but the most abundant class (47% of reads) remained Betaproteobacteria based on the presence of *Burkholderia* and *Delftia*.

Surface water at King Kong Cavern had the most diverse community in terms of bacterial classes D = 0.84, H = 3.3). Biers et al. [40] found that coastal waters were dominated by Alphaproteobacteria which represented over 50% of all “hits”, whereas it represented only about 25% in the 1.0 m sample above King Kong Cavern. Twenty-three percent of reads in the 1.0 m King Kong sample were gammaproteobacteria while in the Biers et al. samples they averaged only 14%. Five percent of their hits were cyanobacteria, while *Synechococcus* was only 0.5% of classified reads in surface water above King Kong Cavern.

The data presented here indicate that the microbiome of this karst sink hole/cave system is unique and reflects the physicochemical conditions and microbial process within the cave system as well as the incurrent and excurrent tidal flow. Those processes affect the entire water column in the vicinity of the blue hole.

## Conclusions

The microbial communities of mucoid filaments and water column in the turbidity zone at ebb flow in King Kong Cavern were dominated by the genus *Arcobacter*. *A*. *nitrofigilis* and *Arcobacter* sp. UDC415 together represented 75–90% of all the bacterial reads at depths between approximately 18 m and 34 m and were present at high relative abundance at both shallower and deeper depths. Although *Arcobacter* was the most abundant genus in the mucoid filaments the proportion differed between the 18 meters and 21 meters In KS3, *Arcobacter* represented a smaller portion of the reads while *Desulfocapsa* was greater. Microbial diversity was low (D = 0.17, H = 0.7) in the 18 m to 25 m depth range and increased with depth but was highest in the near surface and upper water column.

The bacterial community composition indicates that the ebb flow from the cavern influences the entire water column. The proportion of bacterial classes in near surface water differs from other coastal ocean waters with higher representation of members of Betaproteobacteria and Gammaproteobacteria.

## Supporting information

S1 Video Video of mucoid filaments and turbid water in King Kong Cavern. The vertical west cliff of the blue hole is seen on the left and the rubble pile from the collapsed cavern is visible on the lower left. The mucoid filaments are prominent. Depth is from 20 m to 25 m.
(MP4)

S2 Video Video of the cave mouth at King Kong Cavern. Depth is about 30 m with a view into the cave portion of this ocean blue hole.
(MP4)

S3 Video Collecting samples in King Kong Cavern. Depth is about 18 m.
(MP4)
